# Factors that Affected Women Undergoing Cryotherapy Following Cancer Screening with Visual Inspection of the Cervix Using Acetic Acid Method

**DOI:** 10.31557/APJCP.2020.21.5.1423

**Published:** 2020-05

**Authors:** Dahlan Napitupulu, Herindita Puspitaningtyas, Khabib Mualim, Ardhanu Kusumanto, Lutfan Lazuardi, Susanna Hilda Hutajulu

**Affiliations:** 1 *Field Epidemiology Training Program, Master of Public Health Postgraduate Program, Faculty of Medicine, Public Health and Nursing, Universitas Gadjah Mada, Yogyakarta, Indonesia. *; 2 *Port Health Office Class II, Jayapura, Papua, Indonesia. *; 3 *Clinical Epidemiology Program, Master of Clinical Medicine Postgraduate Program, Faculty of Medicine, Public Health and Nursing, Universitas Gadjah Mada, Yogyakarta, Indonesia. *; 4 *Temanggung District Health Office, Central Java, Indonesia. *; 5 *Division of Gynecologic Oncology, Department of Obstetrics and Gynecology, Faculty of Medicine, Public Health and Nursing, Universitas Gadjah Mada/Dr Sardjito General Hospital, Yogyakarta, Indonesia. *; 6 *Department of Public Health, Faculty of Medicine, Public Health and Nursing, Universitas Gadjah Mada, Yogyakarta, Indonesia. *; 7 *Division of Hematology and Medical Oncology, Department of Internal Medicine, Faculty of Medicine, Public Health and Nursing, Universitas Gadjah Mada/Dr Sardjito General Hospital, Yogyakarta, Indonesia. *

**Keywords:** Cervical intraepithelial neoplasia/diagnosis, uterine cervical neoplasm/diagnosis, acetic acid, cryotherapy

## Abstract

**Background:**

The Indonesian government has applied the cancer “see and treat” method which involves a visual inspection using acetic acid (VIA), followed by a cryotherapy procedure, to reduce the incidence of cervical cancer. However, compliance with the program is still low in the targeted population. This study aims to see what factors influence women to receive cryotherapy treatment if they have positive VIA result.

**Methods:**

This cross-sectional study was conducted among 356 VIA positive women, aged 30-50 years old, registered at Temanggung District Health Office, Central Java, Indonesia between March 29 and April 31, 2018. Data on whether subjects underwent cryotherapy, their demographic profile, education, knowledge about cryotherapy, and family support were collected in a direct interview using a structured questionnaire. A statistical analysis was carried out to observe the influence of all the variables on subjects’ decisions on cryotherapy.

**Results:**

In our study, 217 women (60.69%) received cryotherapy, while 139 women (39.04%) did not. Among all the variables analyzed, the factors affecting the subjects’ likelihood to undergo cryotherapy are their knowledge about cervical cancer and screening (PR=0.776;95%CI=0.660-0.913;p=0.003), their residences distance from health centre (PR=0.795;95%CI=0.650-0.971;p=0.016), permission from their family (PR=0.675;95%CI=0.556-0.820;p=0.018), and being accompanied by their family (PR=0.824;95%CI=0.700-0.970;p=0.026). Age, marital status, occupation, and education background did not show a significant correlation with the women’s decisions to receive cryotherapy.

**Conclusions:**

Interestingly, the result of our study indicates that women are less willing to undergo the cryotherapy procedure if they have good knowledge about the cryotherapy procedure and its importance in cervical cancer’s prevention. Providing higher quality and more accessible health facilities with cryotherapy services are important in influencing women’s willingness to receive cryotherapy. Family support, in the form of permission given by spouses, and if they accompanied the patient to seek cryotherapy care are observed as factors influencing women’s willingness to have the procedure.

## Introduction

Cervical cancer remains one of the malignancies with a high burden worldwide. There were 569,847 new cases and 311,365 deaths recorded globally in 2018. According to Globocan 2018, it is currently ranked fourth for women and tenth overall with an incidence rate of 13.1 per 100,000 population. Cervical cancer is also the ninth most common cause of death by cancer, with a mortality rate of 6.9 per 100,000 population and a five year survival rate of 38.98%. At present, it is the second most prevalent cancer in the Indonesian population, with an incidence rate of 23.4 per 100,000 population, a mortality rate of 13.9 per 100,000 population, and a five year survival rate of 63.54% (Bray et al., 2018). 

Most cervical cancer carcinogenesis pathways are associated with human papillomavirus (HPV) infection, which is often asymptomatic. Among one million women with this infection, 10% are expected to develop the disease (Basu et al., 2018; Soler et al., 2000). As much as 96% of cervical cancer patients were positive for HPV and 83% of the individuals are infected with HPV 16 or HPV 18 (Nuranna et al., 2012).

This risk of infection mainly occurs in women aged 30 to 40 years old. HPV infection causes dysplasia that may grow into precancerous lesions, confined to the outer lining of the cervical epithelium (carcinoma in situ (CIS)). If not detected or treated immediately, about 1.6% of individuals with CIS lesions will develop into malignant cancer. The development of high-grade squamous intraepithelial lesions (HGSILs) into cancer can occur within 10 to 20 years, although it can only take one or two years in several cases (Deksissa et al., 2015).

Cancer screening has long been observed to be effective in reducing the incidence and mortality of cervical cancer (Landy et al., 2016). Several methods that are suitable for screening in low-resource settings like Indonesia have recently been assessed for their effectiveness. Pap smear techniques are among the basic methods for early detection. These techniques are difficult to apply throughout the country, due to the limited number of anatomical pathology specialists who are capable of determining a diagnosis based on a pap smear examination (Nuranna et al., 2012). Visual inspection of the cervix using acetic acid (VIA) can be an alternative screening procedure for early detection. A VIA examination is done by direct visual inspection of the cervix to observe the presence of cancer resembling lesions. The cervix is then rubbed with a 3-5% acetic acid solution and observed for any elevated or thickened white plaque, which indicates positive results (World Health Organization, 2011). This procedure can be immediately followed with cryotherapy for initial treatment of the pre-cancerous lesions. Such a method has also been proven to be reliable, sensitive and a more affordable alternative to pap smears (Kim et al., 2013; Nuranna et al., 2012).

Ideally, the presence of aceto-white lesions upon VIA examination should be confirmed by a colposcopy procedure to observe neovascularization, one of the signs of neoplasia, indicated by punctuation, mosaic, or coarse vascular changes (Pretorius et al., 2015; Waxman et al., 2017). The finding of pre-cancerous lesions should be followed by performing a punch biopsy on the dense area for histopathological confirmation (Waxman et al., 2017). These chains of procedures are often less effectively performed due to the limited facilities and few pathologists being available, leading to failures in completing the diagnosis and treatment of women with positive initial screening results (Adefuye et al., 2015; Nuranna et al., 2012; Ouedraogo et al., 2018). Several strategies have been created to avoid these risks, including the colposcopy-and-treat approach and screen-and-treat approach (Basu et al., 2017). Treatment without histopathologic or colposcopy verification is considered as the most effective strategy to improve compliance, especially in low-resource settings (Denny et al., 2010, 2005; World Health Organization, 2011). 

The commonly used therapeutic method following a positive screening is the loop electrosurgical excision procedure (LEEP). LEEP is an office-based excisional procedure that uses heat from a high voltage electrical arc between the operating electrode and the tissue to create a vaporization or coagulation effect, hence cutting the targeted tissue. LEEP has the ability to treat occult invasive cancer rather than only removing superficial tissue, as other methods such as ablative therapy do. Another approach that has a relatively close cure rate to LEEP is cryotherapy, an alternative method that can be done under conditions where supporting infrastructure is not available (Khan and Smith-McCune, 2014; Singh et al., 2011). The procedure is done directly by freezing the abnormal tissue area using a cryoprobe, which will freeze the cervix (-60 to -90^o^C) using N_2_O or CO_2_ gas. The frozen tissue will gradually disappear, and the cervical layer will recover to its former condition. Cryotherapy does not cause bleeding and will only result in minimal pain. Common side effects are liquid discharge that can appear for up to two to three weeks after therapy, and a risk of infection during mucosal healing. Although least recommended, the WHO suggests that cryotherapy is one of the important efforts to prevent cervical cancer. Importantly, this procedure can be done immediately after the simple, reliable visual inspection of the cervix using acetic acid (VIA) method. Immediate cryotherapy after a VIA examination has become a potential means to reduce the number of untreated patients. If effectively performed on premalignant cervical lesions, its cure rate at varying degrees of CIN is 84-97% (Lewis et al., 2011; Luciani et al., 2008; Nene et al., 2008; Soler et al., 2000; World Health Organization, 2011). 

Several attempts to improve screening coverage with subsequent early treatment for cervical lesions that had previously been done in Indonesia showed unsatisfactory results. The see-and-treat model that was carried out with 970 VIA positive women in Jakarta showed that only 654 (67.4%) of the participants underwent cryotherapy (Nuranna et al., 2012). The Cervical and Breast Cancer Prevention Program (CEPAP) that was conducted in Karawang district showed that 7 of 17 health facilities only performed screening on less than 20% of the target population. Of all the VIA positive subjects, 83.1% (678 of 816 VIA positive women) participated in the cryotherapy program. These included 13% (20 individuals) that underwent an immediate procedure and 74% (115 individuals) had it within one month during the last 18 months of the observation period (Kim et al., 2013). 

Information on the factors that may influence VIA positive women to undergo cryotherapy will be useful to the programs that intend to increase the coverage of screening and treatment. However, information on this issue is still very limited. Thus, this study aims to explore the affecting factors, to contribute to the improvement of cervical cancer screening and early treatment in Indonesia.

**Table 1:** Subjects’ Characteristics

Variables	N (%)
Age	
<35 years	158 (44.38)
≥35 years	198 (55.62)
Marital status	
Married	349 (98.03)
Widowed	7 (1.97)
Occupation	
Housewife or house assistant	223 (62.64)
Farmer	62 (17.42)
Civil servant	11 (3.09)
Private employee	36 (10.11)
Other jobs	24 (6.74)
Distance from residence to health facility	
≤2 km	112 (31.46)
>2 km	244 (68.54)
Educational background	
College or university	19 (5.34)
High school	85 (23.88)
Middle school	114 (32.02)
Elementary school	126 (35.39)
No formal education	12 (3.37)
Response to cryotherapy procedure	
Undergo cryotherapy	217 (60.96)
Refuse cryotherapy	139 (39.04)
Knowledge about cryotherapy	
Good	214 (60.11)
Poor	142 (39.89)
Family permission	
Yes	339 (95.22)
No	17 (4.78)
Family accompaniment	
Yes	231 (64.89)
No	125 (35.11)

**Table 2:** Factors Influenced Women to Undergo Cryotherapy

Variable	Cryotherapy			
	Receive cryotherapy	Refuse Cryotherapy	OR	*P*-value
	(N=217)	(N=139)	(95% CI)	
Age				
<35 years	92 (58.2)	66 (41.8)	0.922	0.346
≥35 years	125 (63.1)	73 (36.9)	(0.778-1.092)	
Marital status				
Married	213 (61.0)	136 (39.0)	1.068	0.835
Widowed	4 (57.1)	3 (42.9)	(0.559-2.040)	
Occupation				
Housewife or house assistant	133 (59.6)	90 (40.4)	1.059	0.511
Farmer	49 (79.0)	13 (21.0)	(0.894-1.254)	
Civil servant	5 (45.5)	6 (54.5)		
Private employee	11 (30.6)	25 (69.4)		
Other jobs	19 (79.2)	5 (20.8)		
Distance from residence to health facility				
≤2 km	58 (51.8)	54 (48.2)	0.795	0.016
>2 km	159 (65.2)	85 (34.8)	(0.650-0.971)	
Educational background				
College	10 (52.6)	9 (47.4)	1.303	0.212
High school	31 (36.5)	54 (63.5)	(0.805-2.109)	
Middle school	61 (53.5)	53 (46.5)		
Elementary school	109 (86.5)	17 (13.5)		
No formal education	7 (58.3)	5 (41.7)		
Knowledge about cryotherapy				
Good	117 (54.7)	97 (45.3)	0.776	0.003
Poor	100 (70.4)	42 (29.6)	(0.660-0.913)	
Family permission				
Yes	202 (59.6)	137(40.4)	0.675	0.018
No	15 (88.2)	2 (11.8)	(0.556-0.820)	
Family accompaniment				
Yes	131 (56.7)	100 (43.3)	0.824	0.026
No	86 (68.8)	39 (31.2)	(0.700-0.970)	

## Materials and Methods

We conducted a cross-sectional study at Temanggung District Health Office, Central Java, Indonesia, between March 29 and April 31, 2018. A screening program using VIA examinations, involving 5,323 individuals, was performed in primary health centers within the region during the last six months. We used a simple random sampling technique to select 356 subjects from 2,013 women who had positive results. Patients who were unable to communicate independently with the interviewer were excluded prior to the subjects’ selection. The subjects were local residents and aged between 30-50 years. 

We categorized the subjects according to whether they underwent cryotherapy following a VIA examination or refused to do so. Data on the subjects’ demographic profiles, education, knowledge and awareness about cryotherapy, and their family’s support, were collected to determine the factors influencing the subjects’ decisions related to cryotherapy. Data were obtained by direct interviews with the researcher in an unblinded fashion, using a structured questionnaire to avoid bias. Specifically, the subjects’ knowledge of cervical cancer and the importance of screening and the cryotherapy treatment were assessed using a validated questionnaire. Knowledge was scored and then determined as good when the subjects could answer 75% or more of the questions, and poor when they answered less than 75% of the questions. The study has been approved by the Ethics Committee of the Faculty of Medicine, Public Health and Nursing Universitas Gadjah Mada, Yogyakarta (reference number: KE/FK/0281/EC/2018). 

Following the data’s collection, a statistical analysis was performed using STATA software. A descriptive analysis was performed to present the baseline characteristics of the subjects. A bivariate analysis, using the chi square test, was done to observe the association between cryotherapy and the other factors observed. 

## Results

*Subjects’ characteristics*

The majority of the subjects were women aged ≥ 35 years old (n=198, 55.62%) and married (n=349, 98.03%). The vast majority of subjects had at least an elementary school (n=126, 35.39%) or junior high school education (n=114, 32.02%) with only 19 subjects (5.34%) having studied at university, while 12 subjects (3.37%) did not have any formal education. At the time of the study, the majority of subjects were housewives or household assistants (n=223, 62.6%), while the rest were farmers (n=62, 17.42%) or private employees (n=36, 10.11%).

Most of the study’s subjects resided more than two kilometers from the primary health centers (n=244, 68.54%). Among all the study’s participants, 217 subjects (60.69%) received cryotherapy following a positive VIA result, while 139 subjects (39.04%) refused to undergo a cryotherapy procedure, despite having received information about the procedure and its risk. Family support for women to undergo cryotherapy is shown by the fact that 339 subjects (95.22%) claimed that they received permission from their partners. Another kind of support was shown by the 231 subjects (64.89%) who were accompanied by their spouse or a family member to the primary health centers. Prior to the screening program, all the participants received counseling and information about cervical cancer and screening using VIA, which would be followed by the cryotherapy procedure following a positive result. Two-hundred and fourteen (60.11%) subjects had good knowledge about cervical cancer and the screening method.

*Factors affecting the likelihood of having cryotherapy*

Table 2 displays a bivariate analysis of the factors that influenced participants to have the cryotherapy procedure. Our study showed that knowledge about cervical cancer and screening, distance from their residence to the health facility, and family support was associated with the likelihood to undergo cryotherapy. Interestingly, subjects with a good knowledge of cryotherapy and cervical cancer were 0.776 times more likely, or 1.289 times less likely, to undergo cryotherapy (95% CI=0.660-0.913, p=0.003). Subjects who lived less than two kilometers from health care facilities providing cryotherapy were 1.258 times less likely to undergo cryotherapy (PR= 0.795, 95%CI=0.650-0.971, p=0.016). Subjects who received permission from their families were 1.481 times less likely to have cryotherapy (PR= 0.675, 95%CI=0.556-0.820, p=0.018), while being accompanied by family members was correlated with 0.824 times the likelihood of undergoing cryotherapy (95%CI=0.700-0.970, p=0.026). Age, marital status, occupation, and education background did not correlate with individuals’ willingness to undergo the procedure. 

## Discussion

Despite the fact that several programs have been implemented to decrease the high prevalence of cervical cancer in Indonesia, only a few, limited, studies have explored the coverage of cryotherapy in women with positive VIA results. The evaluation of a cervical cancer screening program conducted in Jakarta, Indonesia, showed only 39% of the VIA positive women received cryotherapy (Kim et al., 2012). In the present study, the proportion of subjects who did not undergo cryotherapy was still high (39.04%). This is quite a contrast with similar studies conducted in Thailand, showing only 1.5% of all the subjects refused immediate cryotherapy following a positive VIA result (Gaffikin et al., 2003), and in Guatemala, where only 1% of the subjects refused (Mathers et al., 2005).

Although the Indonesian government had set a target for cervical cancer screening coverage of 80% by 2013, the screening program only achieved 5% of the targeted population. This situation keeps cervical cancer in the second rank of the most prevalent cancers in the country (Afsah, 2010). Factors such as women’s perception, lack of knowledge and the limitation of available facilities are observed to influence the women’s reluctance to receive cryotherapy services (Kim et al., 2012).

In our study, the subjects’ knowledge of the importance of, and procedures for, cryotherapy has the strongest impact on their willingness to undergo cryotherapy (p=0.003). A previous study in Yogyakarta, Indonesia has shown that there was a lack of knowledge about cervical cancer in our population (Dwi Endarti et al., 2017). Interestingly, our result shows that subjects with better knowledge are generally less willing to receive cryotherapy (PR=0.776, 95% CI=0.660-0.913). This finding is similar to the study done in other part of Indonesia showing that knowledge does not translate into a better attitude and behavior towards cancer prevention (Solikhah et al., 2019). 

Perceived barriers are commonly found in Indonesian women due to a lack of interest, motivation, and concern for cancer-related issues (Solikhah et al., 2019). A previous study showed that the perceived barriers present in women who do not perform routine cervical cancer screening would greatly affect women’s reluctance to receive the procedure. Fear of a bad result, embarrassment, other priorities, a lack of information about the health facilities providing the examination and early treatment, as well as the presence of male health care providers were several barriers observed in the study (Afsah, 2010). Generally, the fear of finding cancer and the presence of male physicians are also factors found to be barriers for women’s compliance in cervical cancer screening programs (Akinlotan et al., 2017).

Another strong influence on the subjects receiving cryotherapy in our study is the subject’s distance from health facilities (PR= 0.795, 95% CI=0.650-0.971, p=0.016). Our result shows that those subjects who live closer to health care facilities have a lower likelihood of undergoing the cryotherapy procedure. 

Closer and accessible health care facilities providing VIA and cryotherapy services are thought to be the solution to increase cervical cancer screening and early treatment in Indonesia. Previous studies have indicated a relationship between the distance and availability of health service facilities with increased awareness and willingness of women in the community to access the VIA service. The ability to treat precancerous lesions at primary health care facilities, without needing sophisticated equipment and highly specialized personnel is one of the advantages of cryotherapy following VIA (Luciani et al., 2008). The provision of VIA services and cryotherapy, trained personnel, and supporting equipment in primary health facilities is a good solution to increase the coverage of screening to prevent the further development of cervical cancer (Phongsavan et al., 2011).

A significant impact has been seen in Jakarta, Indonesia, where there is increased participation in screening using VIA after the service became available in health facilities near to where people live, compared to when it was only available at hospitals far away from them (Kim et al., 2013). Furthermore, a study in Laos also found that the reason why subjects with positive VIA results refused cryotherapy treatment on the same day is because they were required to travel to a health facility far from their location, which supports this result (Phongsavan et al., 2011).

It has been generally accepted that location is an important factor in health-seeking behavior. However, previous studies suggested that the geographic placement of new health care facilities is not as important as the cost of the service, and the perception that the health facility provides high-quality preventive, diagnostic and therapeutic services. A study performed in Bo, Sierra Leone, showed that people prefer the health services offered in higher-level health facilities, even when a lower-level facility is available closer to their location. This happens because they prefer to get treated by a more reputable provider, in a facility with more advanced services, while health facilities closer to them may only provide limited services (Fleming et al., 2016).

Family support, in the form of permission (p=0.018) and being accompanied (p=0.026) also influenced the subjects’ decisions about undergoing cryotherapy in our study. A study in Laos showed that delays in cryotherapy treatment were associated with the fact that women had to ask for their partners’ permission (Phongsavan et al., 2011). In fact, the majority of our subjects obtained their partners’ approval, indicating their support and sufficient awareness of the importance of disease prevention. Nevertheless, the subjects in our study who received permission were less likely to receive cryotherapy. Women usually find difficulty in explaining the importance of cryotherapy to their partner, leading to a reluctance to undergo the procedure (Afsah, 2010).

The rejection of medical procedures can be influenced by the different perceptions and beliefs of certain ethnic or socioeconomic groups (Bernstein et al., 2018). Considering that Indonesia is a developing country with a diverse cultural background and economical status, further observations are needed to determine whether these factors also play a role in affecting people’s awareness and acceptance of cryotherapy procedures in our local setting. A study in West Java showed that husbands play a big role in health promotion, including encouraging women to participate in cancer screening and early prevention (Widiasih et al., 2016; Widiasih and Nelson, 2018). In Indonesian culture, a husband usually holds authority in a family, thus his opinion significantly influences a woman’s decision. Women’s lack of skill at explaining to their husbands, and the husbands’ lack of knowledge about screening and cryotherapy, also hinder the women’s participation in cervical cancer prevention (Kim et al., 2012; Widiasih and Nelson, 2018). Local studies observed that most Indonesian men perceived cancer as a fatal and costly disease in women, and that they should actively fight against it (Widiasih and Nelson, 2018).

The current study has some limitations. It was quantitatively designed and only measured the strength of the influencing factors from the subject’s perspective. The family members’ perspective toward cryotherapy and cervical cancer screening and prevention may serve as a target for further study. This may provide useful information to strengthen the role of the family in increasing women’s participation in screening and prevention programs. Another limitation of this study was its inability to provide information about the supporting instruments and health providers needed to perform cryotherapy. This aspect is subject to further investigation. Indeed, the availability and accessibility of cryotherapy as a first-line treatment is highly essential in low resource settings, as stated by a previous study (Lewis et al., 2011). 

In the present study, we showed that most individuals with VIA positive results were willing to receive cryotherapy in an effort to prevent cervical cancer. Among the subjects we studied, knowledge about the importance of cryotherapy and its procedures, the distance from a health facility, family permission and if they accompanied the patient influenced the refusal behavior. Our study’s results suggested that increasing the subjects’ awareness and family involvement are crucial to improving the success of the cervical cancer prevention program.
